# GSK3β-mediated Keap1-independent regulation of Nrf2 antioxidant response: A molecular rheostat of acute kidney injury to chronic kidney disease transition

**DOI:** 10.1016/j.redox.2019.101275

**Published:** 2019-07-17

**Authors:** Minglei Lu, Pei Wang, Yingjin Qiao, Chunming Jiang, Yan Ge, Bryce Flickinger, Deepak K. Malhotra, Lance D. Dworkin, Zhangsuo Liu, Rujun Gong

**Affiliations:** a Institute of Nephrology, The First Affiliated Hospital of Zhengzhou University, Zhengzhou, 450052, China; b Division of Kidney Disease and Hypertension, Brown University School of Medicine, Providence, RI, 02903, United States; c Division of Nephrology, University of Toledo College of Medicine, Toledo, OH, 43614, United States; d Denison University, Granville, OH, 43023, United States; e Department of Medicine, University of Toledo College of Medicine, Toledo, OH, 43614, United States; f Department of Physiology and Pharmacology, University of Toledo College of Medicine, Toledo, OH, 43614, United States

**Keywords:** Chronic kidney disease, Renal tubular cells, Antioxidant, Atrophy, Lithium

## Abstract

Transition of acute kidney injury (AKI) to chronic kidney disease (CKD) represents an important cause of kidney failure. However, how AKI is transformed into CKD remains elusive. Following folic acid injury, mice developed AKI with ensuing CKD transition, featured by variable degrees of interstitial fibrosis and tubular cell atrophy and growth arrest. This lingering injury of renal tubules was associated with sustained oxidative stress that was concomitant with an impaired Nrf2 antioxidant defense, marked by mitigated Nrf2 nuclear accumulation and blunted induction of its target antioxidant enzymes, like heme oxygenase (HO)-1. Activation of the canonical Keap1/Nrf2 signaling, nevertheless, seems intact during CKD transition because Nrf2 in injured tubules remained activated and elevated in cytoplasm. Moreover, oxidative thiol modification and activation of Keap1, the cytoplasmic repressor of Nrf2, was barely associated with CKD transition. In contrast, glycogen synthase kinase (GSK)3β, a key modulator of the Keap1-independent Nrf2 regulation, was persistently overexpressed and hyperactive in injured tubules. Likewise, in patients who developed CKD following AKI due to diverse etiologies, like volume depletion and exposure to radiocontrast agents or aristolochic acid, sustained GSK3β overexpression was evident in renal tubules and coincided with oxidative damages, impaired Nrf2 nuclear accumulation and mitigated induction of antioxidant gene expression. Mechanistically, the Nrf2 response against oxidative insult was sabotaged in renal tubular cells expressing a constitutively active mutant of GSK3β, but reinforced by ectopic expression of dominant negative GSK3β in a Keap1-independent manner. *In vivo* in folic acid-injured mice, targeting GSK3β in renal tubules *via* conditional knockout or by weekly microdose lithium treatment reinstated Nrf2 antioxidant response in the kidney and hindered AKI to CKD transition. Ergo, our findings suggest that GSK3β-mediated Keap1-independent regulation of Nrf2 may serve as an actionable therapeutic target for modifying the long-term sequelae of AKI.

## Highlights

• AKI to CKD transition involves sustained GSK3β overactivation and impaired Nrf2 response in injured renal tubules.
• Microdose lithium rectifies GSK3β overactivity in the kidney, reinstates Nrf2 response and hinders AKI to CKD transition.
• GSK3β-mediated Keap1-independent regulation of Nrf2 is a novel therapeutic target for modifying long-term sequelae of AKI.

## 1 Introduction

Acute kidney injury (AKI) has been traditionally regarded as a self-limiting mild disease that reverses rapidly and spontaneously. Indeed, following AKI, most patients recover their baseline kidney function and only a minority requires hemodialysis after their initial hospital discharge [1,2]. However, a growing body of evidence recently demonstrates that the number of patients with incomplete renal recovery might have been underestimated [[3], [4], [5], [6]]. In agreement, epidemiologic studies suggest that AKI *per se* is an independent and important risk factor for subsequent development of chronic kidney disease (CKD), featured by progressive loss of kidney function and renal fibrosis [[7], [8], [9]]. The pathogenic mechanisms by which AKI transforms into CKD remains to be defined, but there is evidence suggesting that failed tubular recovery, possibly due to very severe damage or impaired repair, may play a central role [8,[10], [11], [12]]. In cases that tubules fail to recover, tubular epithelial cells remain in a state of dedifferentiation and tubular atrophy ensues, resulting in exuberant production of diverse fibrogenic and proinflammatory cytokines that via paracrine signaling induce inflammatory infiltration, activation and proliferation of fibroblasts, and accumulation of extracellular matrix in kidney interstitium. A number of pathogenic pathways have been implicated in incomplete recovery of tubular epithelial cells from AKI, including aberrant cell cycle arrest, hypoxia, lingering inflammation and others [10,[13], [14], [15], [16], [17], [18]]. Regardless of the various etiologies of AKI, like acute ischemia reperfusion injuries, nephrotoxicity or urethral obstruction, all these pathways are both causes and consequences of excessive oxidative stress due to overproduction of reactive oxygen and nitrogen species (RONS). As a common denominator of both AKI and CKD, oxidative stress has been demonstrated to be responsible for failed tubular recovery after AKI and transition of AKI to CKD [11,19,20].

To protect against the oxidative insults of free radicals and RONS, mammalian cells have evolved an intricate antioxidant self-defense system that maintains redox homeostasis and cellular integrity and enables adaptation to stresses. Central to this self-protective antioxidant defense is NF-E2-related factor (Nrf2), a cap-n-collar basic-region leucine zipper nuclear transcription factor that mediates the primary cellular defense against the cytotoxic effects of oxidative stress [21,22]. As the master transcriptional regulator of the cellular redox state, Nrf2 transactivates a broad range of molecules involved in antioxidation, detoxification, cell survival, anti-inflammatory response, and more. It has become as an attractive therapeutic target for a number of diseases [[23], [24], [25], [26]], including AKI or CKD, whereas its role in AKI to CKD transition remains largely unknown.

The Nrf2 antioxidant response is a complex and highly orchestrated pathophysiological process that is under the regulation by a myriad of signaling pathways. In a state of low oxidative stress, Nrf2 is sequestered in the cytoplasm and associated with an inhibitor protein called Kelch-like enoyl-CoA hydratase-associated protein 1 (Keap1) [22,27]. Upon its activation triggered by oxidative stress, Nrf2 dissociates from Keap1 and subsequently translocates into the nucleus, where Nrf2 recognizes and binds to a conserved antioxidant response element (ARE) and results in transcriptional activation of cytoprotective genes encoding phase II detoxifying enzymes, like heme oxygenase (HO)-1 [21,28]. In addition to Keap1-dependent Nrf2 regulation, Keap1-independent regulatory pathways also play a key role in governing Nrf2 response at a delayed/late phase [29]. Many of these pathways are integrated and funneled down to a decision point where the Nrf2 response is either continued or ceased. Glycogen synthase kinase (GSK) 3β has emerged as the integration point and is pivotal in switching off the self-protective antioxidant stress response after injury by facilitating nuclear exclusion and degradation of Nrf2, thus dictating the magnitude and duration of the stress elicited Nrf2 antioxidant response [30,31]. The presence of oxidative damages in the kidney during AKI to CKD transition suggests that the antioxidant self-defense might have been impaired [10]. However, whether this is due to a dysregulated Nrf2 signaling is unknown. This study examined the role of Keap1 dependent and Keap1-independent regulation of Nrf2 in AKI to CKD transition in a murine model of folic acid induced kidney injury and in cultured renal tubular epithelial cells *in vitro*.

## 2 Material and methods

### 2.1 Animal experiment design

Animal studies were approved by the institutional Animal Care and Use Committees, and they conform to the US National Institutes of Health Guide for human care and use of laboratory animals and the ARRIVE guidelines.

#### 2.1.1 Murine model of folic acid-induced nephropathy

Male C57BL/6 mice aged 10 weeks received an intraperitoneal injection of folic acid (250 mg/kg, Sigma-Aldrich, St. Louis, MO, USA) dissolved in sodium bicarbonate (300 mM) or vehicle as previously described [32]. On day 0, 3, 7 and 14 after folic acid insult, a small volume of blood (less than 0.1 ml) was collected by submandibular bleeding using a lancet. All mice were euthanized on day 28 and blood and organs were collected for further examination. Serum creatinine levels were evaluated retrospectively. Ten mice were treated with folic acid. Vehicle-treated mice served as control.

In a separate study, male C57BL/6 mice aged 10 weeks received an intraperitoneal injection of folic acid as elaborated above. On day 3, blood samples were collected by submandibular bleeding and serum creatinine levels screened. Mice with serum creatinine levels ranging between 2.0 to 2.4 mg/dl were selected for the follow-up study and others were euthanized. On day 3, 7, 14, and 28 after folic acid insult, 5 to 10 mice were randomly picked and euthanized. Blood and organs were collected for further investigation.

#### 2.1.2 Experimental design for mice with renal tubule-specific GSK3β knockout

Mice with renal tubule-specific GSK3β knockout (KO) and the control littermates (Con) were generated by mating floxed-GSK3β mice with γGT.Cre mice according to the protocol established in previous studies [33]. Male Con and KO mice at 10 weeks of age received an intraperitoneal injection of folic acid (300 mg/kg). On day 3, blood samples were collected by submandibular bleeding and serum creatinine levels screened. Mice with serum creatinine levels ranging between 2.0 to 2.4 mg/dl were retained for the follow-up study and others were euthanized. On day 0, 3, 7, 14, and 28 after folic acid insult, five random mice from each of the Con and KO groups were euthanized. Blood and organs were collected for further investigation.

#### 2.1.3 Lithium treatment for murine models of folic acid-induced nephropathy

To optimize the regimen of lithium therapy, male C57BL/6 mice aged 10 weeks received an intraperitoneal injection of folic acid (250 mg/kg) as elaborated above. On day 7 after injury, mice were randomized to receive a subcutaneous injection of microdose LiCl (40 mg/kg) or an equal molar amount (1 mEq/kg) of sodium chloride as saline as reported before [34]. Subsequently, 3 to 4 mice from each treatment group were euthanized every other day. Kidney samples were collected for immunoblot analysis of inhibitory phosphorylation of GSK3β at serine 9.

After the regimen of weekly microdose lithium treatment was ascertained, a separate experiment was carried out. In brief, male C57BL/6 mice aged 10 weeks received an intraperitoneal injection of vehicle or folic acid (250 mg/kg) as described above. Beginning day 7 after injury, mice were randomly assigned to the following groups to receive diverse treatments: (1) FA + NaCl group: mice were given a subcutaneous injection of sodium chloride (1 mEq/kg) as saline once a week. (2) FA + LiCl group: mice were given a subcutaneous injection of LiCl (40 mg/kg) once a week. (3) FA + LiCl + Trig group: Besides once a week LiCl treatments, mice received an intraperitoneal injection of trigonelline (Trig, 1 mg/kg) every other day. On day 0, 3, 7 and 14 after folic acid insult, a small volume of blood (less than 0.1 ml) was collected by submandibular bleeding. On day 28, all mice were euthanized; blood and kidney samples were collected for further investigation. Four mice were randomly assigned to each group.

### 2.2 Human kidney biopsy samples

Human research participants were not specifically recruited for this study. The use of unidentified human biopsy specimens in this study conformed to the ethical guidelines of the 1975 Declaration of Helsinki and was approved by the Institutional Review Board of Zhengzhou University. Excessive or discarded kidney biopsy samples have been banked at the Renal Pathology Laboratories of the First Affiliated Hospital of Zhengzhou University and formalin-fixed and paraffin-embedded archived specimens were used. Included in this study were kidney biopsy specimens from patients with histology-proven CKD subsequent to a documented antecedent AKI due to diverse etiologies, including use of iodinated radiocontrast media, extracellular fluid volume contraction or ingestion of aristolochic acid-containing herbs. In addition, morphologically normal post-transplant protocol biopsy tissues from kidney transplant patients with normal kidney function were used as controls for complete recovery from the AKI. Additional kidney specimens without histomorphologic lesions were procured from kidneys discarded for transplantation due to vascular anomalies and served as normal controls.

### 2.3 Renal morphology assessment and immunohistochemistry analysis

Formalin-fixed and paraffin-embedded kidney tissues were prepared in 3 μm sections. For general kidney histology, sections were processed for periodic acid-Schiff (PAS) or Masson Trichrome staining to estimate the severity of kidney injury. One observer performed semi-quantitative morphometric analysis in a blinded manner. Alternatively, Image J software was used for computerized morphometric analysis of kidney fibrosis. The average fibrosis area was determined by evaluating five random fields per section. For immunohistochemistry staining, the sections were deparaffinized and rehydrated. After heat-induced epitope retrieval, peroxidase immunohistochemical staining was performed as described before by using primary antibodies for Nrf2 (Abcam, Cambridge, MA), p-Histone H3 (Ser 10) and GSK3β (Cell Signaling Technology, Danvers, MA), HO-1(Santa Cruz Biotechnology, Santa Cruz, CA), and 8-OHdG (Santa Cruz Biotechnology). As a negative control, the primary antibody was replaced by preimmune serum from the same species; no staining occurred.

### 2.4 RNA extraction and semiquantitative reverse transcriptase-polymerase chain reaction (RT-PCR)

Total RNA was extracted from kidney specimens by using the RNeasy kit (Promega, Madison, WI, USA) according to the manufacturer's instruction. The first strand cDNA was prepared using Superscript RT reverse transcriptase (Invitrogen, Carlsbad, CA). Semiquantitative RT-PCR assay of mRNA expression of HO-1, NQO1, Trx1and GAPDH genes was carried out as previously described [35] by using the following primers: *HO-1*, 5′-GGAACTTTCAGAAGGGCCAG-3' (forward) and 5′-GTCCTTGGTGTCATGGGTCA-3' (reverse); *NQO1*, 5′-CCCACAAGGTTGCAGCCGGA-3' (forward), 5′-CGGGCGTCTGCTGGAGTGTG-3' (reverse); *Trx1*, 5′-CGCCGGGCGTGCCAGTTTAT-3' (forward), 5′-TGGCTCCAGAAAATTCACCCACC-3' (reverse); *GAPDH*, 5′-CAATGCCTCCTGCACCACCA-3' (forward), 5′-GATGTTCTGGAGAGCCCCGC-3' (reverse). PCR amplification was conducted for a number of cycles in the linear range as determined in preliminary experiments. PCR products resolved in 1.5–2% agarose gels were photographed under ultraviolet light.

### 2.5 Cell culture and transient transfection

Murine proximal tubular epithelial cells (TKPT cells) were cultured in DMEM/F12 medium that contained 5% Fetal Bovine Serum [36]. The vectors encoding Empty Vector (EV), the haemagglutinin (HA)-tagged constitutively active (S9A) mutant (S9A-GSK3β-HA/pcDNA3) or kinase dead (KD) mutant (K85R-GSK3β-HA/pcDNA3) of GSK3β were transfected to TKPT cells by using Lipofectamine 2000 (Life Technologies, Carlsbad, CA) as previously described [37]. Transfection efficiency was verified by immunofluorescence staining or immunoblotting for HA at 16 h. Then cells were treated with hydrogen peroxide (200 mM), tert-butylhydroquinone (tBHQ, 20 μM) or vehicle for 48 h. Cells were subsequently collected and prepared for Western blot analysis or immunocytochemical staining. The intracellular levels of 8-isoprostane, tripeptide glutathione (GSH) and glutathione disulfide (GSSG) were determined by assaying cell lysates as elaborated before [38,39] using commercial kits as per the manufacture's protocols (Cayman Chemical Company, Ann Arbor, MI, USA). Results were normalized by total protein contents and expressed as relative abundance or GSH/GSSG ratios.

### 2.6 Immunofluorescence staining

Cultured cells or frozen kidney cryostat sections were fixed and processed for fluorescent immunostaining. Samples were stained with primary antibodies against HA or Nrf2, followed by applying the Alexa Fluor-conjugated secondary antibodies (Invitrogen, Carlsbad, CA). As a negative control, primary antibodies were replaced by preimmune serum. For TdT-mediated dUTP nick end labeling (TUNEL) analysis, cells were fixed with 4% paraformaldehyde in phosphate-buffered saline and processed for staining with a TUNEL kit (Promega). Finally, samples were counterstained with 4′,6-diamidino-2-phenylindole (DAPI) or propidium iodide (PI), mounted with Vectashield mounting medium (Vector Laboratories, Burlingame, CA, USA) and visualized by fluorescence microscope (BX43, Olympus, Tokyo, Japan). ImageJ software was used for post processing of the images, e.g. scaling, merging, and colocalization analysis.

### 2.7 Western immunoblot analysis

The cultured cells were lysed and kidney tissues homogenized in radioimmunoprecipitation assay buffer containing protease inhibitors. Nuclear fractions were prepared with the NE-PER kit (Thermo Scientific, Rockford, Illinois, USA) according to the manufacturer's instruction. Protein samples were processed for immunoblot analysis as previously described [40]. The antibodies against Nrf2 and lipocalin-2 were purchased from Abcam, those against GSK3β, p-GSK3β (S9), β-Tubulin and p-Histone H3 (Ser10) were purchased from Cell Signaling Technology, and those against HO-1, nitrotyrosine and histone H3 were acquired from Santa Cruz Biotechnology.

### 2.8 Detection of the oxidized form of Keap1

The oxidized form of Keap1 harboring oxidative thiol modification was examined as previously reported [41]. In brief, kidney specimens were homogenized and cultured cells lysed in radioimmunoprecipitation assay buffer containing protease inhibitors and 20 mM N-ethylmaleimide to stabilize the sulfhydryl moieties in all proteins. Then the disulfide bonds were reduced by treating with 20 mM 2-dithiothreitol (DTT) and afterwards selectively labeled with 50 μM 3-N-maleimido-propionyl biocytin (MPB) after removing the unreacted DTT. To detect Keap1-specific disulfide moieties, the MPB-labeled proteins were immunoprecipitated by Keap1 antibody and immunoprecipitates subjected to immunoblot analysis by detection with horseradish peroxidase-conjugated streptavidin (Sigma-Aldrich).

### 2.9 Statistical analyses

For immunoblot analysis and RT-PCR assay, bands were scanned and the integrated pixel density was determined using a densitometer and the ImageJ analysis program (National Institutes of Health, Bethesda, MD). All *in vitro* studies and immunoblot analyses were repeated three to six times. All data are expressed as mean ± SD or as otherwise indicated. Data from two groups were compared by Student's *t*-test. Statistical analysis of the data from multiple groups was performed by one-way ANOVA followed by LSD or Dunnett's T3 test. *P* < 0.05 was considered to represent a statistically significant difference.

## 3 Results

### 3.1 The long-term renal outcome after folic acid-elicited AKI in mice is contingent on the extent of oxidative damage in renal tubules

**Fig. 1:**
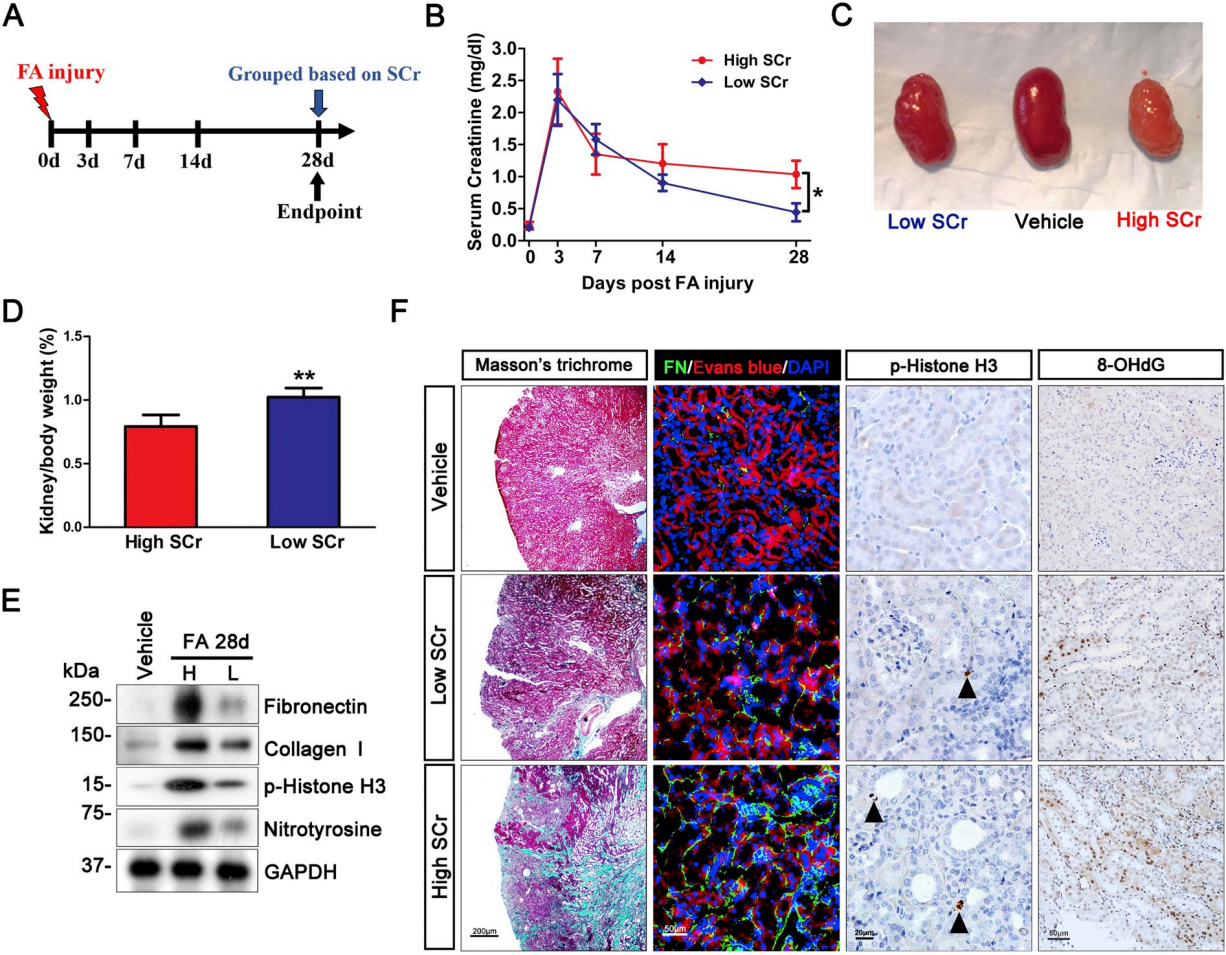
AKI to CKD transition in folic acid-injured mice is contingent on the extent of oxidative damage in renal tubules. Male C57BL/6 mice (n = 10) received an intraperitoneal injection of folic acid (FA, 250 mg/kg) and were followed up for 28 days before sacrifice. On indicated days after folic acid insult, blood was sampled and serum creatinine measured. (A) Schematic diagram illustrates the animal experimental design. (B) Based on each animal's serum creatinine level with reference to their median level on day 28, animals were dichotomized into two subgroups: the low serum creatinine (SCr) or good outcome subgroup and the high serum creatinine or poor outcome subgroup, *P = 0.032 (n = 5). (C) Macroscopic difference in the shape of kidneys procured from the low or high serum creatinine subgroup; (D) Kidney to body weight ratios for the low or high serum creatinine subgroups expressed as percentages of body weights. **P = 0.002 (n = 5); (E) Immunoblot analysis of kidney homogenates for fibronectin (FN), collagen I, phosphorylated histone H3 (p-Histone H3), nitrotyrosine and glyceraldehyde 3-phosphate dehydrogenase (GAPDH). (F) Kidney specimens procured from each group on day 28 were processed for Masson trichrome staining and peroxidase immunohistochemistry staining for phosphorylated histone H3 or 8-hydroxydeoxyguanosine (8-OHdG). Cryosections of kidney tissues were processed for fluorescent immunohistochemistry staining for fibronectin (FN) with Evans blue and 4′,6-diamidino-2-phenylindole (DAPI) counterstaining. . (For interpretation of the references to color in this figure legend, the reader is referred to the Web version of this article.)

To study the pathogenesis of AKI to CKD transition, we employed the murine model of folic acid nephropathy, which recapitulates a disease course featured by AKI during the initial acute phase that lasts for 3–5 days ensued by variable renal recovery and progression to CKD. Mice were followed for 4 weeks and blood samples collected on indicated days (Fig. 1A). It is known that there is inter-individual variation in this model with some mice displaying considerable recovery while others progressing to advanced CKD. In our hands, 28 days after folic acid injection, mice developed a variable degree of CKD. Based on each animal's serum creatinine level on day 28 with reference to their median level, all animals were dichotomically divided into low and high serum creatinine subgroups, respectively corresponding to subgroups with good and poor outcomes. The subgroup with good outcome attained a creatinine level almost comparable to that at baseline. In contrast, the subgroup with poor outcome had proven transition to CKD, as evidenced by the remarkably elevated serum creatinine levels. Shown in Fig. 1B, retrospective time course analysis suggested that serum creatinine levels of the two subgroups began to diverge on day 14, but were essentially comparable on day 0 to day 7 during the acute phase, implying that both subgroups of mice sustained AKI at a similar extent but afterwards experienced distinct disease courses. Consistent with the differences in kidney function as reflected by serum creatinine levels, kidney gross morphology and histology are markedly different between the two subgroups. Kidneys collected from the subgroup with good outcome demonstrated a nearly normal shape with minimal granular surface (Fig. 1C), normal kidney to body weight ratios (Fig. 1D) and negligible extracellular matrix accumulation in tubulointerstitium, as revealed by immunblot analysis of kidney homogenates for fibronectin and collagen I (Fig. 1E) and by Masson trichrome staining and fluorescent immunohistochemistry staining for fibronectin (Fig. 1F). In contrast, kidneys from the poor outcome subgroup displayed a shrunk kidney shape with prominent granular surface and pale color (Fig. 1C) and reduced kidney/body weight ratios (Fig. 1D), in parallel with a typical renal histology of progressive CKD, characterized by tubular atrophy, interstitial fibrosis and inflammation. Moreover, shown by immunoblot analysis (Fig. 1E) and immunohistochemistry staining (Fig. 1F) for phosphorylated histone H3 at serine 10, a hallmark of cell cycle arrest at the G2/M phase, the poor outcome subgroup as compared with the good outcome subgroup demonstrated more renal tubular cell growth arrest, which has been recently implicated in kidney fibrogenesis and AKI to CKD transition. Oxidative stress is a common denominator in the pathogenesis of both AKI and CKD. Indeed, immunoblot analysis of kidney homogenates for nitrotyrosine (Fig. 1E), a marker of protein oxidation, and immunohistochemistry staining for 8-hydroxydeoxyguanosine (8-OHdG, Fig. 1F), an oxidized nucleoside of DNA, revealed that the subgroup with poor outcome had significantly more oxidative kidney injury than the good outcome subgroup.

### 3.2 AKI to CKD transition in folic acid-injured mice is associated with persistent oxidative injury

**Fig. 2:**
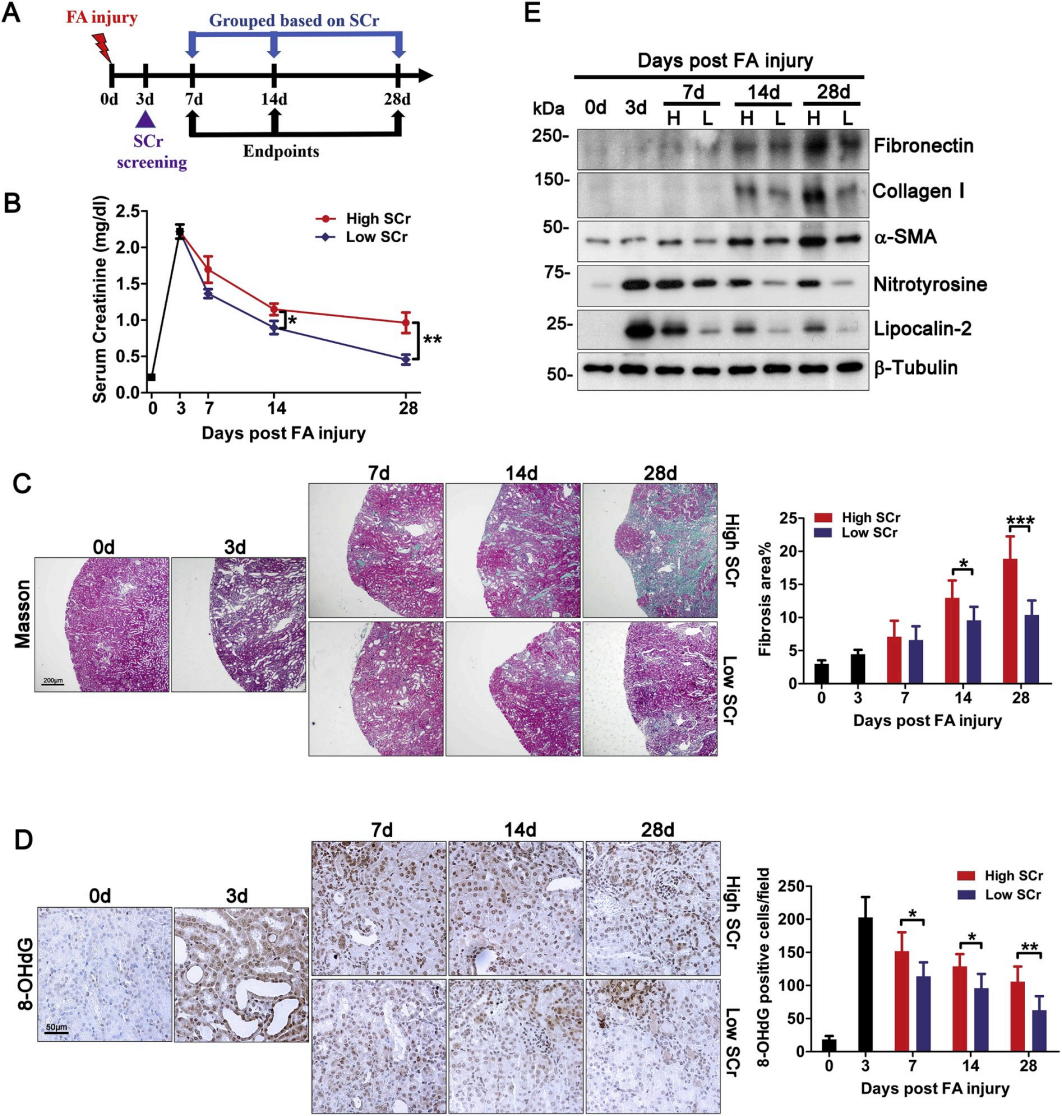
Transition of folic acid-elicited AKI to CKD in mice is associated with persistent oxidative injury. Male C57BL/6 mice received an intraperitoneal injection of folic acid as elaborated in Fig. 1. On day 3, serum creatinine (SCr) levels were screened. Mice with serum creatinine levels ranging between 2.0 to 2.4 mg/dl were selected for the follow-up study in order to assure near uniform AKI, which was further validated by histology of kidney procured from random mice (n = 5) on day 3. Mice were then followed until indicated endpoints. (A) Schematic diagram illustrates the animal experimental design. (B) On post-injury day 7, 14 or 28, 10 mice were randomly picked and euthanized. Based on each animal's serum creatinine level with reference to their median level on indicated time points, mice were divided into low and high serum creatinine subgroups for each time point. *P = 0.032, **P = 0.007 (n = 5). (C) Kidney specimens procured on indicated time points from each group were processed for Masson trichrome staining, followed by computerized morphometric estimation of percentage area of kidney fibrosis by evaluating five random fields per section. *P = 0.022, ***P = 0.000 (n = 4–5). (D) Kidney specimens procured on indicated time points from each group were processed for peroxidase immunohistochemistry staining for 8-OHdG, followed by absolute counting of positive cells per microscopic field by evaluating five random fields per section. *P = 0.013 on day 7, *P = 0.029 on day 14, **P = 0.005 on day 28 (n = 4–5). (E) Immunoblot analysis of kidney homogenates for fibronectin, collagen I, α-smooth muscle actin (α-SMA), nitrotyrosine, lipocalin-2 and β-tubulin.

The different severity of AKI may contribute to variable long-term outcomes [11]. To rule out this confounding factor and to validate the above findings, we next carried out a separate prospective study of murine models of folic acid nephropathy. In order to select mice with near uniform AKI, mouse serum creatinine was measured 3 days after folic acid injury and only mice with a creatinine level between 2.0 and 2.4 mg/dl were included for the follow-up. On day 7, 14 and 28, 10 random mice were sacrificed and dichotomically divided into low and high serum creatinine subgroups based on each animal's serum creatinine level with reference to their median level (Fig. 2A). Shown in Fig. 2B, beginning day 14, the low and high serum creatinine subgroups started to exhibit significantly different serum creatinine levels, indicative of different recovery of kidney function. This was in line with the difference between the two subgroups in renal histologic signs of CKD transition, such as interstitial fibrosis and tubular atrophy as shown by Masson trichrome staining (Fig. 2C). Semi-quantitative morphometric analysis indicated that renal histologic changes started to diverge as early as day 7 but the difference between the two subgroups did not reach statistical significance until day 14. Morphologic findings were further validated by immunoblot analysis of kidney homogenates for molecular markers of renal fibrosis, including α-SMA, collagen I and fibronectin (Fig. 2E). Moreover, oxidative insults in renal parenchyma were also strikingly different between the two subgroups, as revealed by immunohistochemistry staining for 8-OHdG (Fig. 2D), which was mainly located to renal tubules. During the acute phase on day 3, oxidative injury was abundant, but it progressively receded in the low serum creatinine subgroup. In contrast, the high serum creatinine subgroup demonstrated a persistent oxidative damage. Morphometric analysis revealed that the difference in oxidative damage between the two subgroups reached statistical significance after day 7. This finding was further corroborated by immunoblot analysis of kidney homogenates for nitrotyrosine (Fig. 2E). The persistent oxidative stress in the high serum creatinine subgroup was consistent with a sustained renal tubular injury, marked by prolonged expression of lipocalin-2 as detected by immunoblot analysis of kidney homogenates (Fig. 2E). On the contrary, in the low serum creatinine subgroup, lipocalin-2 expression and renal tubular injury progressively subsided after AKI.

### 3.3 Nrf2 antioxidant response is impaired during AKI to CKD transition in folic acid-injured mice and associated with sustained GSK3β hyperactivity in renal tubules

**Fig. 3:**
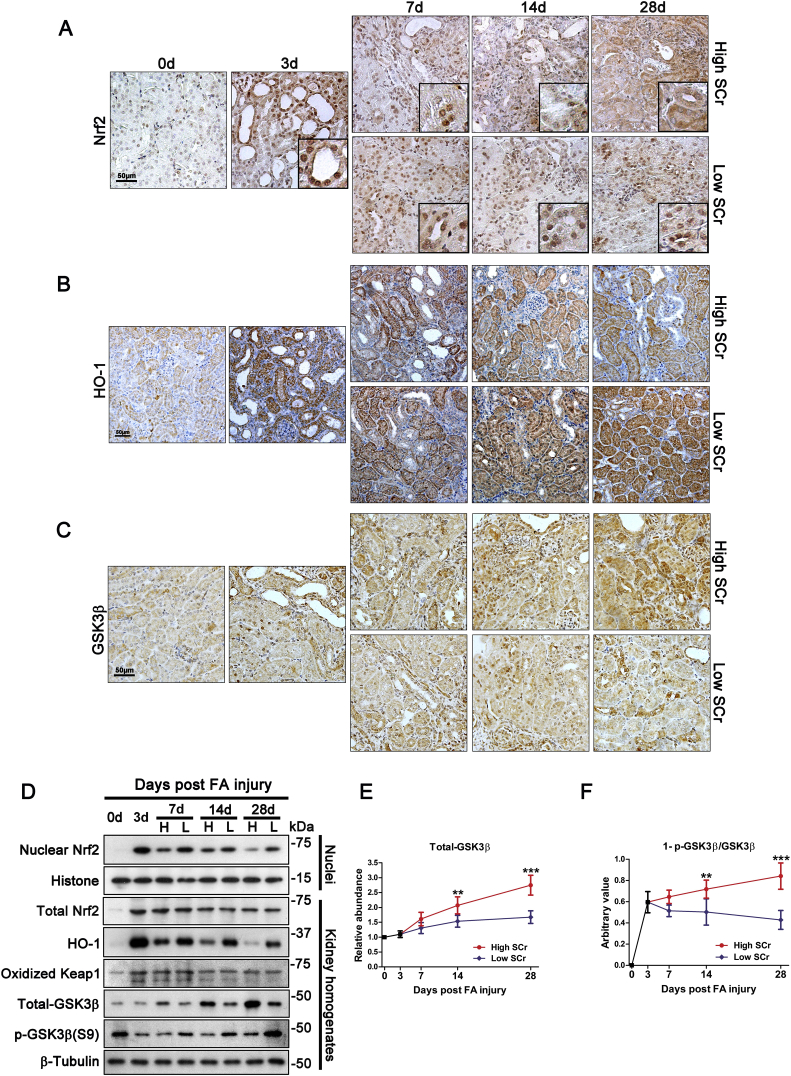
Nrf2 antioxidant response is blunted, as evidenced by impaired Nrf2 nuclear accumulation, in renal tubules during AKI to CKD transition in folic acid-injured mice, concomitant with GSK3β hyperactivity. Mice were treated as elaborated in Fig. 2. Kidney specimens procured on indicated time points from low or high serum creatinine (SCr) subgroups were processed for peroxidase immunohistochemistry staining for (A) Nrf2, (B) heme oxygenase (HO)-1 and (C) GSK3β. Representative microscopic images were shown. (D) Immunoblot analysis of kidney homogenates or nuclear fractions for indicated molecules followed by densitometric analyses of the relative expression level of total GSK3β or phosphorylated GSK3β at serine 9 residue as normalized to β-tubulin. (E) Relative expression levels of GSK3β in the kidney. **P = 0.008 on day 14 and ***P < 0.01 on day 28 versus the low serum creatinine subgroup (n = 5). (F) Arbitrary value of 1-p GSK3β/GSK3β in the kidney for each subgroup. **P = 0.009 on day 14 and ***P < 0.01 on day 28 versus the low serum creatinine subgroup (n = 5).

Upon oxidative stress, mammalian cells immediately resort to the Nrf2/ARE signaling as the primary and only measure for antioxidant response and self-defense. Thus, the persistent renal oxidative injury observed during AKI to CKD transition implies that Nrf2 antioxidant response may be undermined. To test this hypothesis, kidney specimens were processed for immunohistochemistry staining for Nrf2 and its target antioxidant enzyme, HO-1. Shown in Fig. 3A, during the acute phase of folic acid injury (day 3), there was a drastic Nrf2 induction in injured renal tubules that was predominantly located to the nuclei (Fig. 3A), denoting an activated Nrf2 antioxidant response. Accordingly, expression of HO-1 in injured tubules was elevated (Fig. 3B). Nrf2 nuclear accumulation and HO-1 induction persisted in renal tubules in the low serum creatinine subgroup. In stark contrast, in the high serum creatinine subgroup that developed CKD transition, Nrf2 nuclear accumulation in renal tubules was diminished despite an increase in cytoplasmic Nrf2, thus indicative of an impaired Nrf2 response (Fig. 3A). This coincided with a blunted HO-1 induction in renal tubules (Fig. 3B). These morphologic findings were further validated by immunoblot analysis of whole kidney homogenates and nuclear fractions for HO-1 and Nrf2 respectively. Shown in Fig. 3D, despite a comparable induction of total Nrf2 in whole kidney homogenates prepared from the low and high serum creatinine subgroups following folic acid-elicited AKI, nuclear expression of Nrf2 was markedly diminished in the high serum creatinine subgroup that developed CKD transition. The impaired Nrf2 nuclear accumulation in renal tubules during AKI to CKD transition was unlikely attributable to an aberrant Keap1-dependent regulation of Nrf2 signaling, because renal expression of oxidized Keap1 was consistently comparable between the low and the high serum creatinine subgroups (Fig. 3D). Recently, burgeoning evidence suggests that GSK3β plays an important role in both AKI and CKD [42]. Moreover, GSK3β is crucial for Nrf2 regulation at a delayed/late phase for Nrf2 nuclear exit and subsequent cease of antioxidant response. As such, it is conceivable to speculate whether GSK3β is involved in the diminished Nrf2 retention during AKI to CKD transition. To test this notion, immunohistochemistry staining for GSK3β was carried out. Shown in Fig. 3C, GSK3β was expressed by renal tubules to a low extent in normal and acutely injured kidney. This pattern and intensity of renal GSK3β expression were negligibly altered in the low serum creatinine subgroup. In contrast, in the high serum creatinine subgroup, there was a progressively augmented expression of GSK3β in renal tubules. This finding was further corroborated by immunoblot analysis of kidney homogenates followed by densitometric analysis (Fig. 3D and E). As a constitutively active kinase, the activity of GSK3β is determined by the inhibitory phosphorylation at the serine 9 residue. Under physiologic condition (i.e. day 0), there was a basal level of GSK3β phosphorylation at serine 9, as shown by immunoblot analysis of kidney homogenates (Fig. 3D). This phosphorylation was suppressed during the AKI phase, but variably reinstated at the chronic phase of folic acid nephropathy. To estimate the relative activity of GSK3β in the kidney, the value of 1-p-GSK3β/GSK3β was estimated based on densitometric analysis of immunoblots (Fig. 3F) and revealed a progressive correction of GSK3β hyperactivity in the low serum creatinine subgroup but a sustained GSK3β overexpression and hyperactivity in the high serum creatinine subgroup.

### 3.4 GSK3β hyperactivity in renal tubules coincides with impaired Nrf2 antioxidant response in patients with AKI to CKD transition

**Fig. 4:**
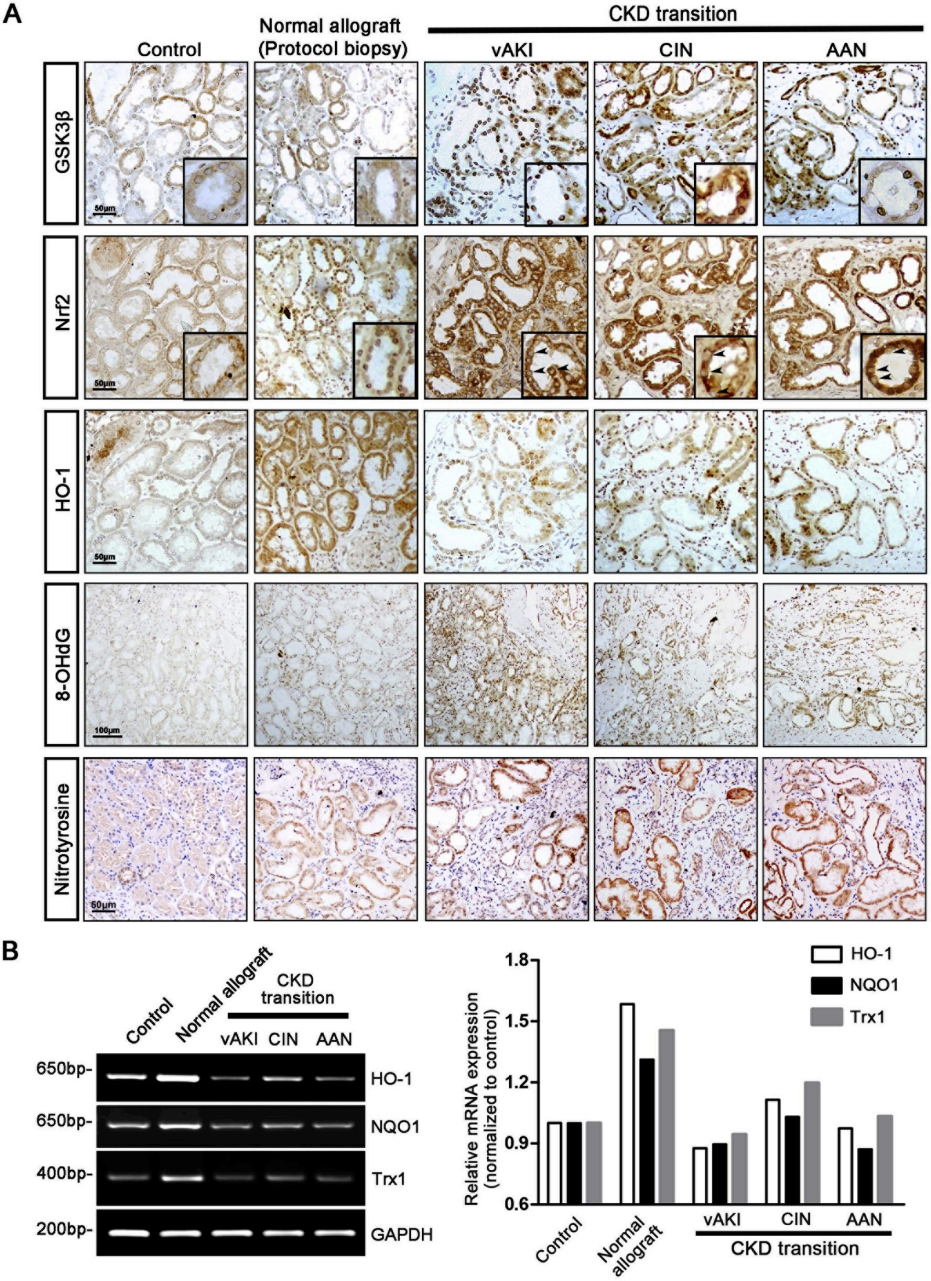
Impaired Nrf2 antioxidant response coincides with GSK3β hyperactivity in renal tubules in human patients with AKI to CKD transition (A) Sections of archived formalin-fixed paraffin-embedded human kidney specimens were processed for peroxidase immunohistochemistry staining for indicated molecules. Included in this study were normal kidney tissues from kidneys discarded for transplantation due to vascular anomalies (Control), morphologically normal post-transplant protocol biopsy tissues from allograft kidneys (Normal allograft), or kidney biopsy specimens procured from patients with histology-proven chronic kidney disease subsequent to a documented antecedent acute kidney injury caused by radiocontrast agents (CIN), contraction of extracellular fluid volume (vAKI) or aristolochic acid-containing herbs (AAN). Representative microscopic images were shown. Highlighted in the inserts are Nrf2 and GSK3β staining in the same tubules in consecutive kidney sections. Note that despite an increased cytoplasmic Nrf2 expression in renal tubules in kidney tissues that had AKI to CKD transition, nuclear Nrf2 expression is markedly diminished in renal tubules (arrow heads). This is concomitant with GSK3β overexpression and hyperactivity in the same tubules as shown by staining of consecutive sections. (B) Assay of mRNA expression of indicated genes in kidney tissues by RT-PCR. GAPDH serves as the internal control gene. The right panel represents densitometric data of the RT-PCR results.

To ascertain if impaired Nrf2 response is also present in human AKI to CKD transition and associated with GSK3β hyperactivity as well, we next examined kidney biopsy specimens from patients with progressive CKD that was developed after a documented antecedent episode of AKI due to diverse etiologies, including administration of iodinated radiocontrast media, contraction of the extracellular fluid volume and ingestion of aristolochic acid-containing herbs. Additional kidney specimens without histomorphologic lesions were procured from kidneys discarded for transplantation due to vascular anomalies and served as normal controls. Because all allograft kidneys are inevitably subjected to ischemia/reperfusion AKI during kidney transplantation, as such, morphologically normal post-transplant protocol biopsy tissues from kidney transplant patients with normal kidney function were used as controls of complete recovery from the AKI. Indeed, minimal oxidative damage, marked by 8-OHdG and nitrotyrosine staining, was noted in protocol biopsy tissues derived from normal allograft kidneys (Fig. 4A). This coincided with a functional Nrf2 response, marked by nuclear accumulation of Nrf2 and HO-1 induction that was associated with normal GSK3β expression detected in the same renal tubules in the consecutive section, and a striking induction of mRNA expression of Nrf2 target genes like *HO-1*, *NQO1* and *Trx1* in the kidney, as estimated by RT-PCR (Fig. 4B). In stark contrast, kidney tissues from patients who developed CKD transition after a previously proven AKI were intensely positive for 8-OHdG and nitrotyrosine staining, indicative of prominent oxidative stress. In parallel, HO-1 induction was blunted in renal tubules and Nrf2 nuclear accumulation was diminished despite abundant Nrf2 located to the cytoplasm, concomitant with GSK3β overexpression in the same renal tubules, as shown by immunohistochemistry staining of consecutive sections (Fig. 4A). This was associated with a blunted induction of mRNA expression of Nrf2 target genes, like *HO-1*, *NQO1* and *Trx1* in the kidney (Fig. 4B).

### 3.5 Keap1-independent GSK3β modulation of the Nrf2 antioxidant defense in renal tubular epithelial cells upon injury

**Fig. 5:**
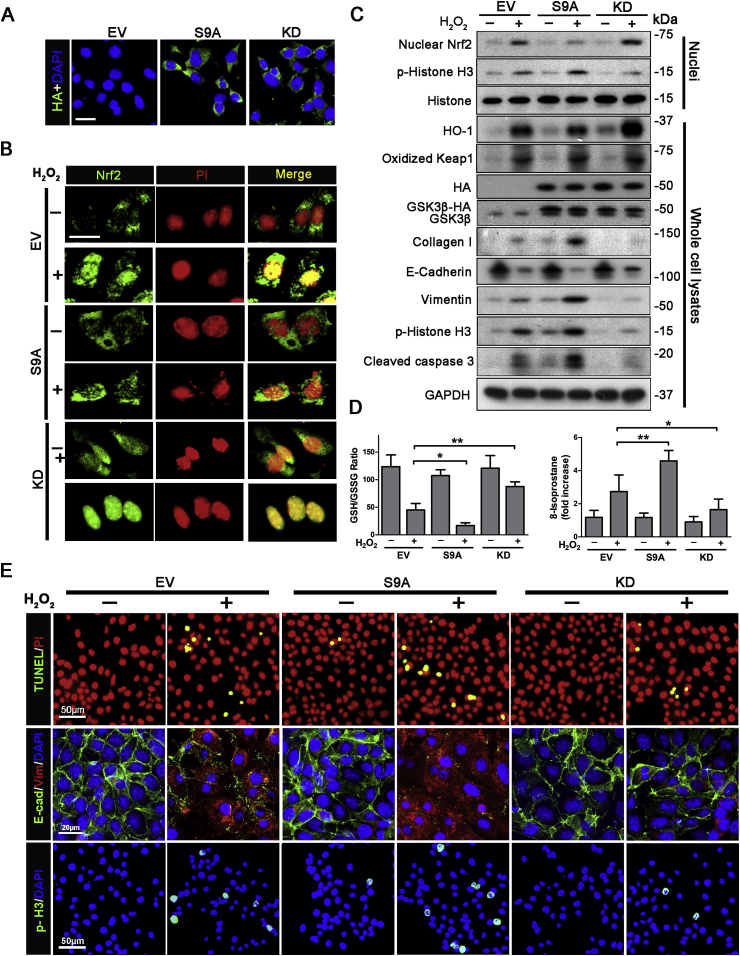
GSK3β dictates the Nrf2 antioxidant defense in renal tubular epithelial cells upon injury in a Keap1-independent mode. TKPT cells were subjected to liposome-mediated transient transfection with vectors encoding HA-conjugated dominant-negative kinase-dead (KD) or constitutively active (S9A) mutant of GSK3β or the empty vector. After transfection for 16 h, cells were injured with hydrogen peroxide (200 mM) for 48 h. (A) Cells were processed for fluorescence immunocytochemistry staining for HA with 4′,6-diamidino-2-phenylindole (DAPI) counterstaining, which demonstrates that the transfection efficiency was >80%. Bar = 20 μm. (B) After hydrogen peroxide injured, cells were fixed and processed for fluorescence immunocytochemistry staining for Nrf2 with propidium iodide (PI) counterstaining. Representative microscopic images were shown. Bar = 20 μm. (C) After hydrogen peroxide injured, cell lysates and nuclear fractions were prepared and subjected to immunoblot analysis for indicated molecules. (D) Measurement of the GSH/GSSG ratios and 8-isoprostane levels in cell lysates. *P < 0.05, **P < 0.01 (n = 3). (E) After hydrogen peroxide injury, cells were fixed and processed for terminal deoxynucleotidyl transferase (TdT) dUTP nick-end labeling (TUNEL) or fluorescence immunocytochemistry staining for E-cadherin (E-cad), vimentin (Vim) or phosphorylated histone H3 (p-H3) with DAPI or PI counterstaining. Representative microscopic images were shown.

To explore if a possible cause effect relation exists between GSK3β hyperactivity and impaired Nrf2 response in renal tubules, the activity of GSK3β in cultured renal tubular epithelial cells was manipulated by ectopic expression of a haemagglutinin (HA)-conjugated dominant-negative kinase-dead (KD) mutant of GSK3β, or an HA-conjugated constitutively active mutant (S9A) of GSK3β that is impervious to inhibition, followed by oxidative insults elicited by hydrogen peroxide exposure. Immunofluorescence staining and immunoblot analysis for HA revealed a satisfactory and equal transfection efficiency (>80%) (Fig. 5A and C). In empty vector (EV)-transfected cells, hydrogen peroxide injury induced some Nrf2 nuclear accumulation (Fig. 5B), consistent with a spontaneous Nrf2 antioxidant self-defense. Immunoblot analysis demonstrated an increased nuclear fraction of Nrf2 and an amplified expression of HO-1 (Fig. 5C). These effects were largely abrogated in cells expressing S9A, but enhanced in KD expressing cells. The GSK3β regulated Nrf2 antioxidant response was paralleled by corresponding changes in cellular redox state, marked by the levels of 8-isoprostane and the GSH/GSSG ratios (Fig. 5D). To this end, hydrogen peroxide injury induced the levels of 8-isoprostanes and reduced GSH/GSSG ratios, indicative of an oxidative stress. This effect was augmented in S9A-expressing cells but blunted in KD-expressing cells. As a key regulator of Nrf2 activity, Keap1 is however unlikely involved in GSK3β regulation of Nrf2 antioxidant response, since S9A- and KD-expressing cells expressed oxidized Keap1 to comparable magnitudes either under basal condition or after injury (Fig. 5C). Indeed, knockdown of Keap1 barely altered the blunted nuclear accumulation of Nrf2 and HO-1 induction in S9A-expressing cells upon hydrogen peroxide injury. In contrast, knockdown of β-TrCP, which is a key factor related to degradation of GSK3β-phosphorylated Nrf2 [31,51], considerably protected Nrf2 activation and preserved HO-1 induction in S9A-expressing cells exposed to hydrogen peroxide (Supplementary Fig. S1). Oxidative stress triggered by hydrogen peroxide resulted in striking cytopathic changes in renal tubular cells, including apoptosis and cell cycle arrest, as detected by TUNEL staining and immunofluorescent staining for phosphorylated histone H3 (Fig. 5E). Cytological findings were further confirmed by immunoblot analysis of cell lysates for activated caspase 3 and of nuclear extracts for phosphorylated histone H3 (Fig. 5C). Moreover, as shown by immunofluorescent staining and immunoblot analysis, hydrogen peroxide injury resulted in *de novo* expression of vimentin, a mesenchymal-specific intermediate filament, and drastic loss of E-cadherin, which are typical signs of tubular cell dedifferentiation, a cellular process critical for CKD transformation. Furthermore, production of extracellular matrix collagen I by renal tubular cells was up-regulated following hydrogen peroxide injury, indicative of a fibrogenic effect. The hydrogen peroxide elicited tubular cell apoptosis, growth arrest, dedifferentiation and extracellular matrix synthesis were reinforced in S9A expressing cells but attenuated in KD expressing cells, in agreement with the difference in Nrf2 response. To determine if the GSK3β regulated Nrf2 response is also applicable to other types of oxidative stress, experiments were repeated by using tBHQ, oxidative insult inducer different from hydrogen peroxide. Shown in Supplementary Fig. S2, tBHQ treatment triggered significant Nrf2 nuclear accumulation in empty vector (EV)-transfected cells, concomitant with HO-1 induction, suggesting a spontaneous Nrf2 antioxidant self-defense. These effects were blunted in cells expressing S9A but potentiated in KD-expressing cells.

### 3.6 Conditional ablation of GSK3β in renal tubules reinforces Nrf2 antioxidant response in a Keap1 independent mode and mitigates AKI to CKD transition in folic acid-injured mice

**Fig. 6:**
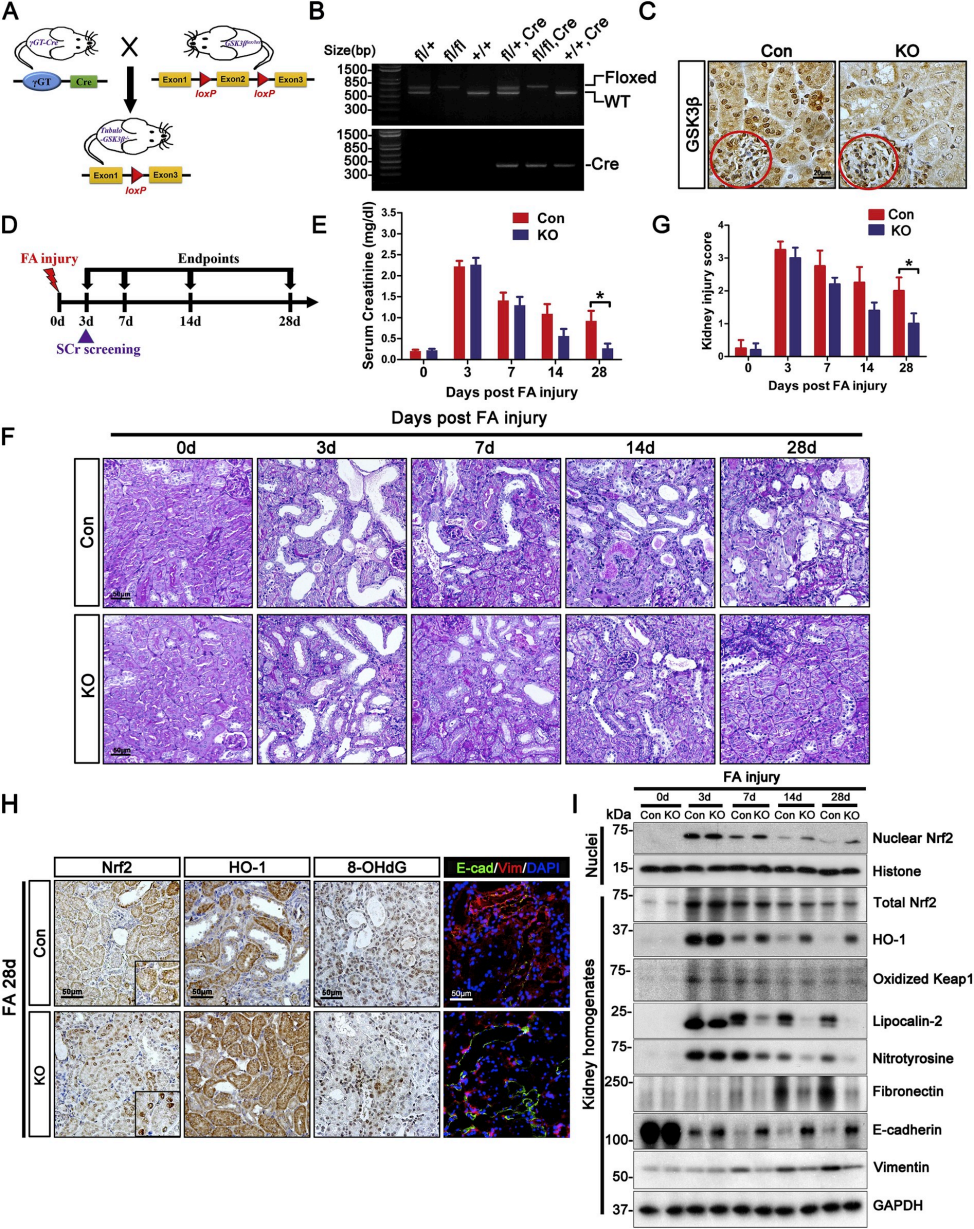
Conditional knockout of GSK3β in renal tubules restores Nrf2 antioxidant response and attenuates AKI to CKD transition in folic acid-injured mice. (A) Schematic diagram depicts the Cre-loxP gene recombination strategy to target the GSK3β gene selectively in renal tubules in adult mice. By crossing the GSK3β-floxed mice with transgenic mice expressing Cre driven by the γ-glutamyl transpeptidase (γGT) promoter, the knockout (KO) mice were bred. Littermates with floxed GSK3β but no Cre transgenes served as control (Con). (B) PCR genotyping of tail DNA from a litter of transgenic mice for Cre and wild-type or floxed GSK3β. (C) Kidney tissues procured from male KO and Con mice at 10 weeks old were prepared for peroxidase immunohistochemistry staining for GSK3β. Note that GSK3β expression in KO kidney was substantially ablated in renal tubules but completely preserved in glomeruli (red circles). (D) Schematic diagram illustrates the animal experimental design. Male KO and Con mice received an intraperitoneal injection of folic acid (300 mg/kg). On day 3, serum creatinine (SCr) levels were screened. In order to study animals with near uniform AKI, mice with serum creatinine levels ranging between 2.0 to 2.4 mg/dl were selected for the follow-up. Mice were then followed until indicated endpoints. (E) Serum creatinine levels of the KO and Con mice on indicated post-injury days. *P = 0.034 (n = 5). (F) Kidney specimens procured on indicated time points from KO and Con groups were prepared for periodic acid-Schiff staining. Representative microscopic images were shown. (G) Semi-quantitative kidney injury score was assessed based on periodic acid-Schiff staining by evaluating five random fields per kidney section. *P = 0.033 (n = 4–5). (H) Kidney specimens procured from KO and Con groups on post-injury day 28 were prepared for peroxidase immunohistochemistry staining for Nrf2, HO-1 or 8-hydroxydeoxyguanosine (8-OHdG) and for dual color fluorescent immunohistochemistry staining for E-cadherin (E-cad) and vimentin (Vim) with 4′,6-diamidino-2-phenylindole (DAPI) counterstaining. (I) Immunoblot analysis of kidney homogenates or nuclear fractions for indicated molecules. (For interpretation of the references to color in this figure legend, the reader is referred to the Web version of this article.)

To substantiate the causal relationship between GSK3β hyperactivity and the impaired Nrf2 response during AKI to CKD transition *in vivo*, mice with conditional knockout of GSK3β (KO) specifically in renal tubules were employed. In agreement with what we previously reported [33], GSK3β was ablated in these mice by the Cre/loxP gene targeting system (Fig. 6A and B) at postnatal mature stage selectively in renal tubules without affecting glomerular expression of GSK3β, as shown by immunohistochemistry staining (Fig. 6C). KO mice together with the sex-matched control littermates were exposed to folic acid insult. It has been reported that loss of GSK3β may variably affect AKI [43]. Thus, in order to study animals with near uniform AKI, mice were given a higher dose of folic acid (300 mg/kg, i.p.) and serum creatinine measured 3 days after folic acid injury. Only mice with a creatinine level between 2.0 and 2.4 mg/dl were included for the follow-up (Fig. 6D). Uniform AKI was assured by comparable serum creatinine levels between KO and control mice (Fig. 6E) and further confirmed by an equal degree of renal histologic injury as evidenced by PAS staining and semi-quantitative kidney injury scoring of kidney specimens procured from random mice in each group 3 days after folic acid injury (Fig. 6F and G). Four weeks after folic acid injury, serum creatinine levels in KO mice were markedly lower than control littermates (Fig. 6E), denoting an improved recovery of renal function. This was associated with ameliorated histologic signs of chronic renal injuries (Fig. 6F), such as interstitial fibrosis, tubular atrophy and inflammation, as shown by PAS staining and estimated by kidney injury scoring. This trend toward a significant difference in serum creatinine and kidney injury score between KO and control mice was noted as early as day 14. These findings infer that loss of GSK3β in renal tubules promotes recovery of renal function and hinders AKI to CKD transition. In parallel, Nrf2 antioxidant response in the injured kidney, marked by Nrf2 nuclear accumulation and HO-1 induction in renal tubules, was prominently enhanced in KO mice. This was concomitant with diminished oxidative damages of renal tubules, as evidenced by 8-OHdG staining, and mitigated tubular cell dedifferentiation, marked by loss of E-cadherin and *de novo* expression of vimentin (Fig. 6H). Morphologic findings were further validated by immunoblot analysis of kidney homogenates (Fig. 6I). The attenuated AKI to CKD transition in KO mice seems to be primarily consequent to a reinforced Nrf2 activity, because KO kidneys displayed much more Nrf2 nuclear accumulation than control kidneys since day 7 after folic acid injury despite similar levels of total Nrf2 in Con and KO kidneys. Subsequently, kidney oxidative damage and tubular injury, marked respectively by renal expression of nitrotyrosine and lipocalin-2 (Fig. 6I), were consistently attenuated in KO kidneys, associated with lessened deposition of extracellular matrix like fibronectin. Keap1 was unlikely involved in the reinforced Nrf2 activity and lessened CKD transition in KO kidneys, since renal expression of oxidized Keap1 was comparable between KO and control mice throughout the follow-up.

### 3.7 Delayed targeting of GSK3β by microdose lithium reinstates Nrf2 antioxidant responses and improves the long-term renal outcome of folic acid-induced AKI in mice

**Fig. 7:**
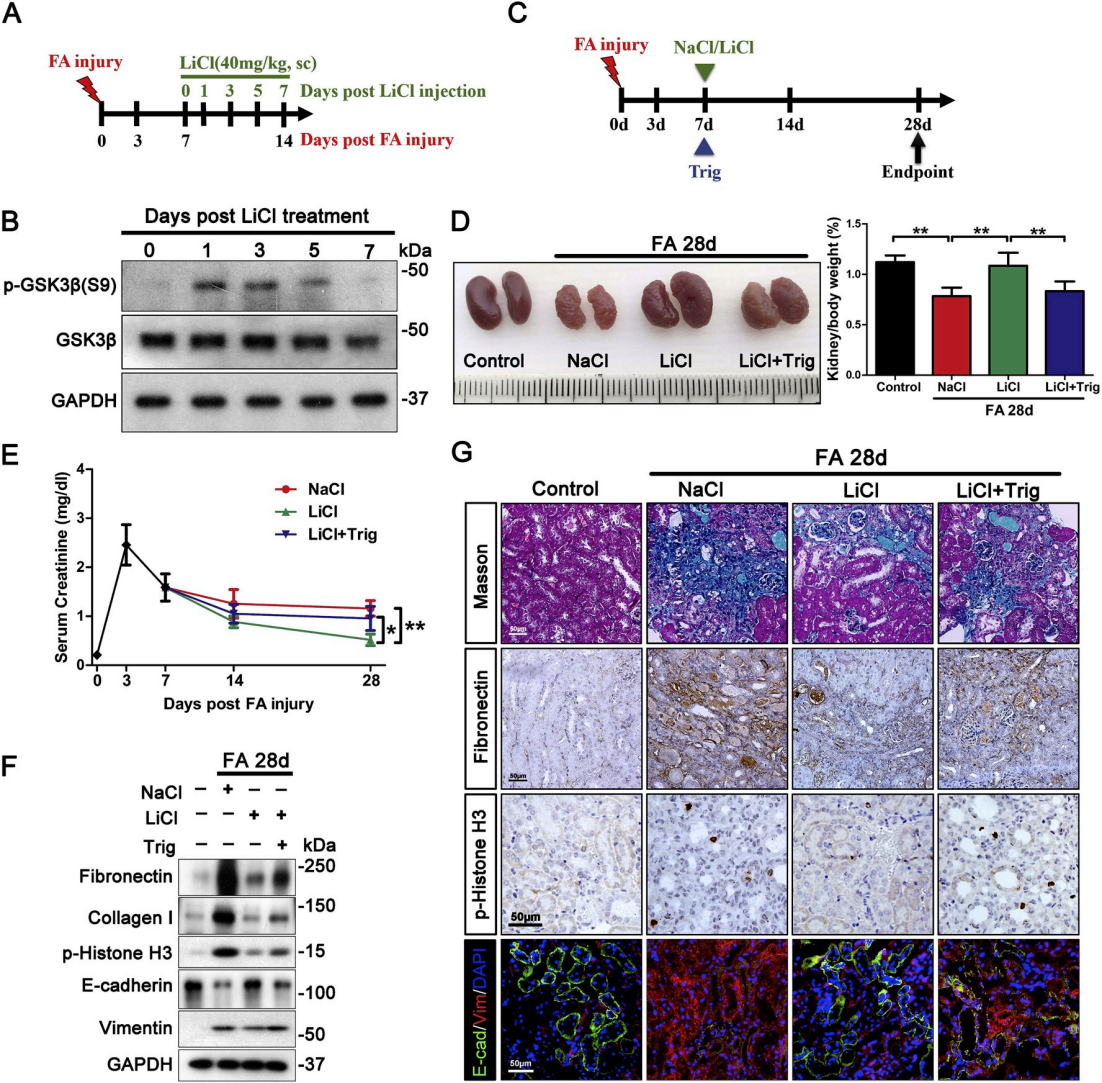
Delayed once a week treatment with microdose lithium hinders AKI to CKD transition in folic acid-injured mice via an Nrf2 dependent mechanism. (A) Schematic diagram illustrates the animal experimental design to optimize the regimen of lithium therapy for mice with folic acid (FA) nephropathy. Male mice received an intraperitoneal injection of folic acid (250 mg/kg) as elaborated in Fig. 1. On day 7 after injury, mice were randomized to receiving a subcutaneous injection of LiCl (40 mg/kg) or an equal molar amount (1mEq/kg) of sodium chloride as saline. Subsequently, 3 to 4 mice from each treatment group were euthanized every other day. (B) Kidney specimens were collected for immunoblot analysis for indicated molecules. (C) Schematic diagram illustrates the animal experimental design to test the efficacy of the delayed weekly microdose lithium treatment on transition of folic acid-induced AKI to CKD in mice. A separate group of male mice received an intraperitoneal injection of folic acid (250 mg/kg). On day 7 after injury, mice were randomly assigned to the following groups to receive sodium chloride (sc, 1mEq/kg, qw) as saline, LiCl (sc, 40 mg/kg, qw), or trigonelline (Trig, ip, 1 mg/kg, qod) plus LiCl (40 mg/kg, qw) until day 28. (D) Representative photos of kidneys procured from each treatment groups on post-injury day 28 and kidney/body weight ratios expressed as percentages of body weights. **P < 0.01 (n = 4). (E) On indicated days, blood was collected and serum creatinine levels measured. *P = 0.034 LiCl versus LiCl + Trig (n = 4); **P = 0.002 LiCl versus NaCl (n = 4). (F) On day 28 after folic acid injury, mice were sacrificed and kidney specimens were processed for immunoblot analysis for indicated molecules. (G) Kidney specimens procured from each group were prepared for Masson trichrome staining and peroxidase immunohistochemistry staining for fibronectin or phosphorylated histone H3. Cryosections were processed for dual color fluorescent immunohistochemistry staining for E-cadherin (E-cad) and vimentin (Vim) with 4′,6-diamidino-2-phenylindole (DAPI) counterstain. Representative microscopic images were shown. (For interpretation of the references to color in this figure legend, the reader is referred to the Web version of this article.)

**Fig. 8:**
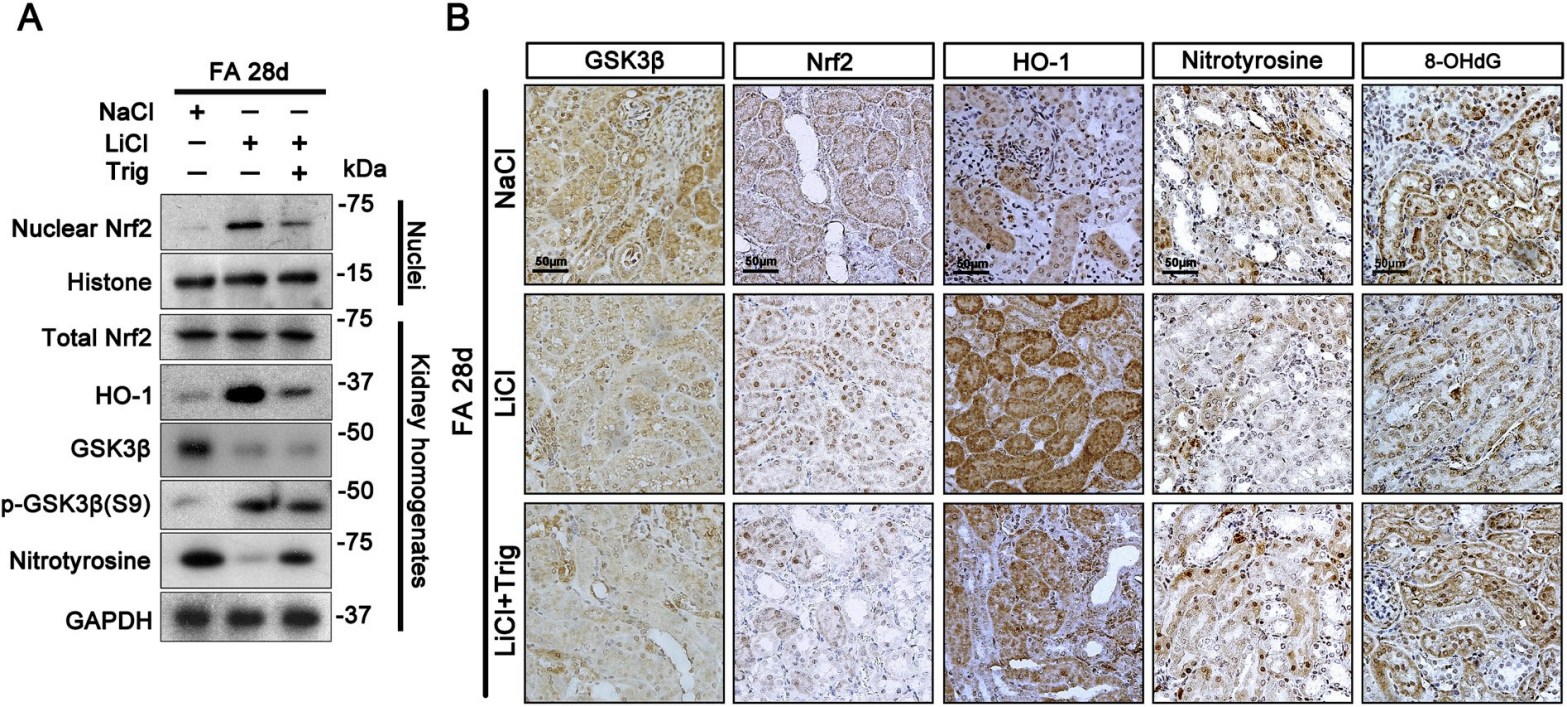
Delayed once a week treatment with microdose lithium overrides GSK3β hyperactivity and reinstates Nrf2 antioxidant response in the kidney, resulting in a lessened oxidative injury and AKI to CKD transition. Mice were treated as described in Fig. 7 (A) On day 28 after folic acid injury, mice were sacrificed and kidney specimens were processed for immunoblot analysis of kidney homogenates and nuclear fractions for indicated molecules. (B) Kidney specimens procured from each group were prepared for peroxidase immunohistochemistry staining for GSK3β, Nrf2, HO-1, nitrotyrosine and 8-OHdG. Representative microscopic images were shown.

To explore if GSK3β-mediated regulation of Nrf2 could be translated into a practical therapeutic target for modifying the outcome of AKI, the efficacy of pharmaceutical targeting of GSK3β in CKD transition was assessed next. Lithium is a standard inhibitor of GSK3β and has been an FDA approved mood stabilizer for the first line treatment of psychiatric disorders for over 50 years [44]. However, psychiatric dose of lithium is considerably high and known to occasionally cause renal adverse effects [45]. Recent studies demonstrated that the dose of lithium to attain effects on peripheral organs is much less than the psychiatric dose [46]. To determine the least optimal dose of lithium that is able to effectively block the activity of GSK3β in the diseased kidney, a pilot study was carried out (Fig. 7A). On day 7 after folic acid injury, mice received a subcutaneous injection of microdose lithium chloride (LiCl, 40 mg/kg) based on our previous experience. Mice were then killed every other day afterwards. An equal molar amount (1 mEq/kg) of sodium chloride (NaCl) as saline served as a control treatment. Shown in Fig. 7B, lithium treatment resulted in a drastic induction of phosphorylation of GSK3β at serine 9, in agreement with the inhibitory activity of lithium on GSK3β. This effect gradually decayed, and by day 7 post lithium treatment, renal expression of phosphorylated GSK3β at serine 9 was almost comparable to that on day 0. Accordingly, a therapeutic regimen of once a week injection of microdose lithium was set up and its efficacy on AKI to CKD transition was tested next. Shown in Fig. 7C, days after folic acid insult, mice were randomized to receiving once a week injection of microdose LiCl or NaCl. Delayed lithium versus sodium treatment significantly improved the gross kidney morphology and serum creatinine levels (Fig. 7D and E), thus denoting a beneficial effect. Indeed, gross shapes of CKD, like kidney shrinkage, pale color, granular surface and the decreased kidney/body weight ratios were all markedly attenuated after lithium therapy (Fig. 7D). Moreover, microscopic histologic signs of CKD transition, including tubular atrophy, interstitial fibrosis and inflammation, were abrogated after lithium treatment, as demonstrated by immunoblot analysis of kidney homogenates for fibronectin and collagen I (Fig. 7F) and verified by Masson trichrome staining and by immunohistochemistry staining for fibronection (Fig. 7G). Furthermore, G2/M cell cycle arrest in renal tubular cells, as detected by immunoblotting or immunohistochemistry staining for phosphorylated histone H3, and tubular cell dedifferentiation, featured by loss of E-cadherin and *de novo* expression of vimentin, were evident in NaCl-treated mice but strikingly diminished after lithium therapy (Fig. 7F and G). This protective effect of lithium was associated with a reinforced Nrf2 antioxidant response in renal tubules, featured by an increase in Nrf2 nuclear accumulation, enhanced HO-1 induction and reduced protein oxidation (nitrotyrosine) and nucleoside oxidation (8-OHdG), as shown by immunoblot analysis of kidney homogenates (Fig. 8A) or by immunohistochemistry staining (Fig. 8B), although renal expression of total Nrf2 was induced by folic acid injury to the same extent in NaCl or LiCl-treated mice. These coincided with a proven inhibitory effect on GSK3β, as evidenced by an augmented inhibitory phosphorylation of GSK3β at serine 9 and a blunted GSK3β induction in renal tubules (Fig. 8A and B). Moreover, the Nrf2 antioxidant response seems to be essential for the above beneficial effects of lithium on attenuating AKI to CKD transition, because simultaneously treatment of mice with trigonelline, a small molecule inhibitor of Nrf2, largely abolished the lithium's effect on improving kidney function, histologic injury and oxidative stress (Figs. 7 and 8).

## 4 Discussion

Epidemiologic evidence proves that AKI *per se* is an independent risk factor for the development of CKD. Even when AKI occurs in the absence of preexisting kidney disease, progressive CKD may still develop [11]. The mechanism underlying this process is, however, poorly understood. Here, we demonstrated that sustained GSK3β hyperactivity following AKI impairs Nrf2 antioxidant self-defense in renal tubules by diminishing nuclear accumulation of Nrf2, thus resulting in persistent oxidative stress that leads to failed tubular recovery and AKI to CKD transition. To the best of our knowledge, our study is the first to prove that the GSK3β mediated Keap1-independent regulation of Nrf2 plays a crucial role in modifying the long-term outcome of AKI.

As the master mediator of the primary cellular detoxification/antioxidant defense, Nrf2 is known to be activated in diverse acute and chronic diseases [[47], [48], [49], [50]]. Nrf2 is regulated by a myriad of cell signaling pathways that involve either Keap1-dependent or Keap1-independent mechanisms. Under basal conditions, Nrf2 is sequestered in the cytoplasm by its intrinsic repressor Keap1 [22]. Many Nrf2 agonists, such as bardoxolone, activate Nrf2 indirectly *via* blocking Keap1 [29]. Once Keap1 is inhibited, Nrf2 will be liberated and translocate into nuclei to initiate the transcription of antioxidant molecules, resulting in a non-specific and systemic activation of the Nrf2 antioxidant response in both stressed and unstressed tissues [51]. This Keap1-dependent mechanism to boost Nrf2 activity, however, seems problematic. Indeed, Keap1 knockout mice do not survive >3 weeks postnatally [52]. In addition, in Keap1-deficient cells, despite an elevated Nrf2 basal activity, the inducibility of the Nrf2-driven genes was hugely blunted upon stress [52]. In consistency, in patients with diabetic kidney disease, bardoxolone therapy worsened albuminuria, caused severe adverse effects, and resulted in excess mortality due to heart failure and cardiovascular events, which prematurely terminated the BEACON trial [53]. Similarly, in a murine model of glomerulopathy, genetic knockdown of Keap1 promoted the constitutive Nrf2 activity but failed to diminish proteinuria [54]. In stark contrast to the Keap1-dependent Nrf2 regulation, the Keap1-independent regulation does not affect the basal activity of Nrf2 but modulates Nrf2 activity principally at a delayed/late phase of Nrf2 response. Thus, it is conceivable that the Keap1-independent regulation may occur mainly in stressed/injured cells where Nrf2 has been activated, rather than in normal cells where Nrf2 is inactive. Moreover, instead of affecting the basal Nrf2 activity, the Keap1-independent regulation of Nrf2 may merely dictate the inducibility, magnitude and duration of Nrf2 response to oxidative stress [29]. A multitude of signaling cascades are able to regulate Nrf2 activity via the Keap1-independent mode, among which GSK3β has emerged as the convergent point [29]. The GSK3β-dictated Nrf2 nuclear exclusion and degradation is pivotal in switching off the Nrf2 response and represents a novel therapeutic target [55,56]. For instance, in patients with chronic hepatitis, inhibition of GSK3β by lithium exerts hepatoprotection through up-regulation of Nrf2 antioxidant response [57]. Likewise, in the kidney, inhibition of GSK3β in glomerular podocytes potentiated Nrf2 response and mitigated podocyte injury following acute adriamycin insult [40]. Here, targeting GSK3β in renal tubules was able to modulate Nrf2 antioxidant response after AKI and affect AKI to CKD transition.

GSK3β is a ubiquitous serine/threonine protein kinase involved in cell signaling pathways responsible for a number of important pathobiologic processes like glycogen biogenesis, redox homeostasis, tissue injury, repair and regeneration [42]. Burgeoning evidence from our and other research groups indicates that GSK3β is overactive in various CKD, including chronic allograft nephropathy, obstructive nephropathy and diabetic nephropathy [42,58]. In agreement, this study showed that GSK3β in mouse kidneys is persistently overactive following folic acid injury, associated with AKI to CKD transition. Folic acid is known to cause AKI in rodents via eliciting oxidative stress subsequent to the formation of luminal crystals in renal tubules or direct toxicity on the tubular epithelium. Oxidative stress is a pivotal pathogenic mediator of folic acid nephropathy at both the early stage of AKI and the later stage of CKD transition [59]. In support of this, antioxidants, like N-acetylcysteine, have been shown to attenuate kidney injury in mice following folic acid insult [60]. In agreement, in our animals, the extent of CKD transition after folic acid elicited-AKI was associated with the magnitude of oxidative damage in tubules, as estimated by measuring protein oxidation (nitrotyrosine) or nucleoside oxidation (8-OHdG). This was at least in part attributable to an impaired Nrf2 antioxidant response, because nuclear accumulation of Nrf2 was apparently diminished, concomitant with a blunted induction of HO-1. This impaired Nrf2 antioxidant response concurrent with GSK3β overexpression was also evident in renal biopsy specimens procured from patients who developed CKD after AKI due to various causes.

The mitigated nuclear accumulation of Nrf2 in renal tubules during AKI to CKD transition is unlikely due to reduced Nrf2 activation, because oxidative thiol modification of Keap1, a triggering process of Nrf2 activation, was comparably detected in kidneys with variable degrees of CKD transition. Rather, some Nrf2-regulating mechanisms are likely involved. GSK3β is known to be able to directly or indirectly *via* Fyn phosphorylate Nrf2 in the nuclei and thereby facilitate Nrf2 nuclear exit [61,62]. In agreement, in cultured renal tubular epithelial cells injured by oxidative insults, spontaneous Nrf2 nuclear accumulation and antioxidant response were reinforced in cells ectopically expressing a dominant negative kinase dead mutant of GSK3β, but lessened in cells expressing a constitutively active GSK3β mutant, without affecting the activity of Keap1. Thus, GSK3β seems to play a pivotal role in dictating the Keap1-independent regulation of Nrf2 response in renal tubules. This was further validated in renal tubule-specific GSK3β knockout mice, which demonstrated a reinforced Nrf2 response and much lessened oxidative injury in renal tubules after folic acid insults, ultimately resulting in a mitigated AKI to CKD transition.

As a redox sensitive kinase, GSK3β activity may be augmented, either through changes in its inhibitory phosphorylation state at serine 9, during the phase of acute injury [36,63], or alternatively via increased expression as seen in chronic diseases, such as diabetes, inflammatory disease and neurodegenerative diseases [[64], [65], [66], [67]]. In CKD, GSK3β overexpression has been observed before [68]. The mechanism remains elusive, but there is evidence suggesting that serine 9 phosphorylation plays a key role. Serine 9 phosphorylation is essential for GSK3β ubiquitination and degradation, and when repressed, will mitigate GSK3β degradation, resulting in GSK3β overexpression [64]. Thus, it is conceivable that excess oxidative stress during AKI suppresses GSK3β inhibitory phosphorylation at serine 9 and augments GSK3β kinase activity, leading to GSK3β accumulation due to reduced ubiquitination and degradation, which promotes GSK3β overactivity, further impairs Nrf2 antioxidant response and exacerbates oxidative injury, resulting in a vicious cycle that drives progressive transition of AKI to CKD.

GSK3β is a druggable kinase that can be selectively inhibited by a number of newly developed small-molecule chemical inhibitors [69,70]. As such, the GSK3β mediated Keap1-independent regulation of Nrf2 activity may be a actionable therapeutic target for hindering post-AKI CKD transition and for improving the long-term outcome of AKI. Among the many selective inhibitors of GSK3β, lithium is the first-generation inhibitor and has been safely used for over a half century as the FDA approved first line mood stabilizer for the treatment of bipolar affective disorders [44,46]. For basic science research, lithium has been commonly used as a standard blockade of GSK3β [71]. The beneficial effect of lithium in various organ systems has been noted for decades. GSK3β inhibition has been responsible for the therapeutic effect of lithium in a number of diseases, such as acute brain injury, chronic neurodegenerative diseases, neutropenia and so forth [72,73]. While long-term (usually >10 years) high-dose lithium therapy primarily for psychiatric disorders is occasionally associated with some kidney adverse effects [74], short term use of microdose lithium has been lately shown by our and other groups to be protective in animal models of kidney diseases, including acute tubular necrosis and podocytopathy [34,75]. Nevertheless, its effect on AKI to CKD transition is unknown. Here, we for the first time provided compelling evidence that once a week microdose lithium, given after AKI, is effective in overriding GSK3β overactivity, restoring Nrf2 antioxidant response and hindering CKD transition.

In summary, sustained GSK3β overactivity in renal tubules after AKI impairs Nrf2 antioxidant response via a Keap1-independent mechanism, resulting in persistent oxidative damages that ultimately lead to CKD transition. Therapeutic targeting of the GSK3β mediated Keap1-independent regulation of Nrf2 in renal tubules by GSK3β knockout or by existing FDA approved agents with GSK3β inhibitory activity, like low-dose lithium is able to mitigate AKI to CKD transition. If confirmed by future clinical trials, the findings in this study may have the potential to lead to novel therapeutic modality for improving the outcome of AKI.

## Author contributions

R.G. devised the conceptual ideas. M.L., P.W., Y.Q., C.J., Y.G. and B.F. carried out the animal and cell culture experiments. Z.L. and M.L. performed the validation experiments on human kidney specimens. M.L., P.W., Y.Q., D.K.M., L.D.D. and R.G. contributed to the discussion and interpretation of the results. R.G. took the lead in writing the manuscript. The entire concept and ownership of this work belong to R.G. All authors approved the final manuscript.

## Conflicts of interest

The authors declare no conflicts of interest.
