# Supplementary material for: GSK3β-mediated Keap1-independent regulation of Nrf2 antioxidant response: A molecular rheostat of acute kidney injury to chronic kidney disease transition
Source: Redox Biol. 2019 Jul 17;26:101275. doi: 10.1016/j.redox.2019.101275 (PMC6669347; doi:10.1016/j.redox.2019.101275)
Supplement: Multimedia component 2 [file mmc2.pdf]

SUPPLEMENTARY MATERIALS

Supplementary Material and Methods

RNA interference (RNAi)

The siRNA oligonucleotides specific for β-TrCP (siβ-TrCP) and Keap1 (siKeap1) were purchased from Santa Cruz Biotechnology (catalogue# sc-37179 and sc-43879) to induce selective knockdown of β-TrCP and Keap1. A scrambled siRNA sequence (5′-GCGAGUAGCGCUAGGAAGUtt-3′) without similarity to any known gene sequences from mouse, rat, or human was used as nonspecific control siRNA for RNAi (siControl). The plasmid encoding Empty Vector (EV) or the haemagglutinin (HA)-tagged constitutively active (S9A) mutant (S9A-GSK3β-HA/pcDNA3) together with one of the above siRNA oligonucleotides were co-transfected into the TKPT cells by using Lipofectamine 2000 (Life Technologies, Carlsbad, CA). Efficiency of lipofectamine-mediated gene-silencing was assessed by immunoblot analysis. Near-complete (~80%) suppression of β-TrCP or Keap1 protein expression was observed in cells transfected with specific siRNA. Cells were then treated with hydrogen peroxide (200 mM) or vehicle for 48 hours followed by immunoblot analysis of cell lysates or nuclear fractions.

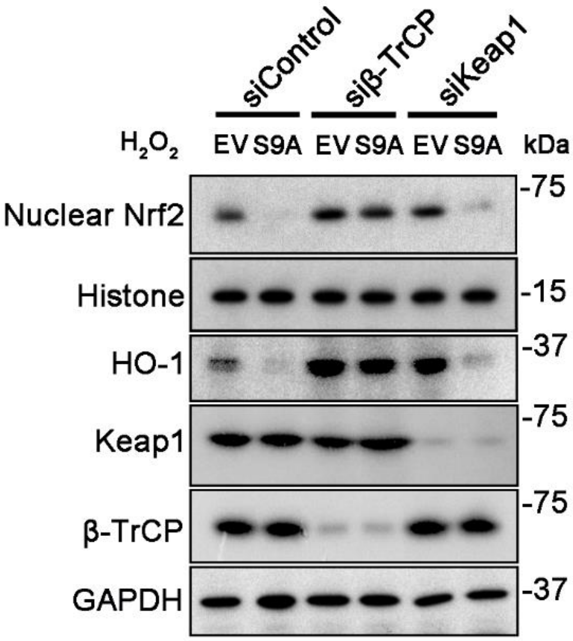

***Supplementary Figure S1*. The effect of β-TrCP or Keap1 knockdown on GSK3β-regulated Nrf2 antioxidant response in renal tubular epithelial cells.**

The empty vector (EV) or the vector encoding haemagglutinin (HA)-tagged constitutively active (S9A) GSK3β mutant (S9A-GSK3β-HA/pcDNA3) together with β-TrCP or Keap1-specific siRNA oligonucleotides or the control siRNA were co-transfected into the TKPT cells by liposome-mediated transient transfection. After transfection for 16 h, cells were treated with hydrogen peroxide (200 mM) for 48 h. Cell lysates and nuclear fractions were prepared and subjected to immunoblot analysis for indicated molecules.

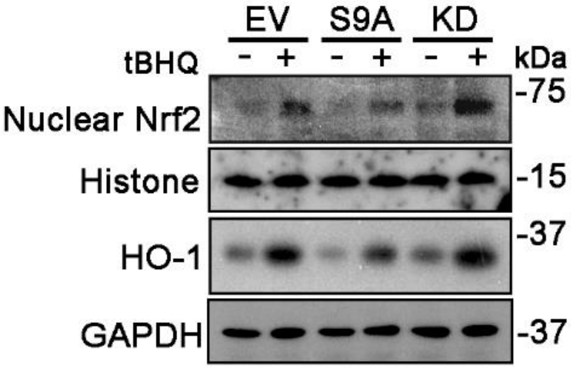

***Supplementary Figure S2*. GSK3β regulates the Nrf2 antioxidant defense in renal tubular epithelial cells upon exposure to tert-Butylhydroquinone.**

TKPT cells were subjected to liposome-mediated transient transfection with vectors encoding HA-conjugated dominant-negative kinase-dead (KD) or constitutively active (S9A) mutant of GSK3β or with the empty vector. After transfection for 16 h, cells were injured with tert-Butylhydroquinone (tBHQ, 20 μM) or vehicle for 48 h. Cell lysates and nuclear fractions were then prepared and subjected to immunoblot analysis for indicated molecules.
